# Supplementary material for: Cognitive Performance during the Development of Diabetes in the Zucker Diabetic Fatty Rat
Source: Cells. 2023 Oct 16;12(20):2463. doi: 10.3390/cells12202463 (PMC10605915; doi:10.3390/cells12202463)
Supplement: Supplementary file 1 [file cells-12-02463-s001.zip › cells-2603107-supplementary.pdf]

Supplementary DATA

Supplementary Figures

Figure S1

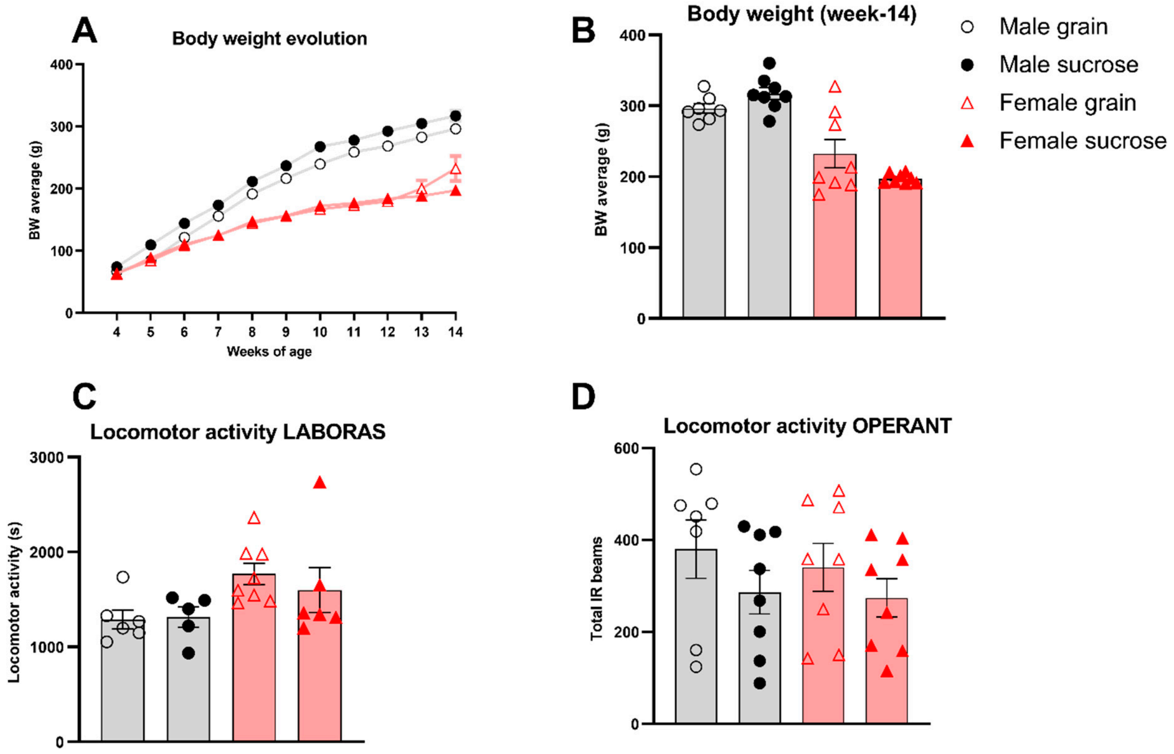

Figure S1. Body weight development and locomotor activity of male and female LE-sucrose and LE-grain rats. (A) Body weight evolution between weeks 4 and 14 of age. We observed a main effect of feeding regime ($F_{(1, 27)\ \text{feeding regime}}$ = 5.99, p=0.021) and an interaction between sex and feeding regime ($F_{(1, 27)\ \text{sex x feeding regime}}$ = 9.49, p=0.0047) during development ($F_{(10, 270)\ \text{sex x feeding regime x weeks}}$ = 1.98, p=0.035). This indicated that LE-sucrose males reached a higher body weight compared to LE-grain males, but no main difference in body weight levels occurred between females. (B) Body weight comparison at week 14 indicated a significant interaction effect ($F_{(1, 27)\ \text{sex x feeding regime}}$ = 7.70, p=0.024) and follow-up posthoc analyses indicated that LE sucrose males showed a trend towards a higher bodyweight compared to LE grain (p=0.077) at week 14 of age. (C) Values represent the mean of 5 sessions of 48h each in the Laboras homecage. A significant effect of sex on locomotor activity in the Laboras homecage was found, with females being more active than males ($F_{\text{sex}(1, 21)}$=6.47, p<0.05). (D) Locomotor activity during the visual discrimination (VD) stage where no significant differences were observed. Values represent the group mean (±SEM).

Figure S2

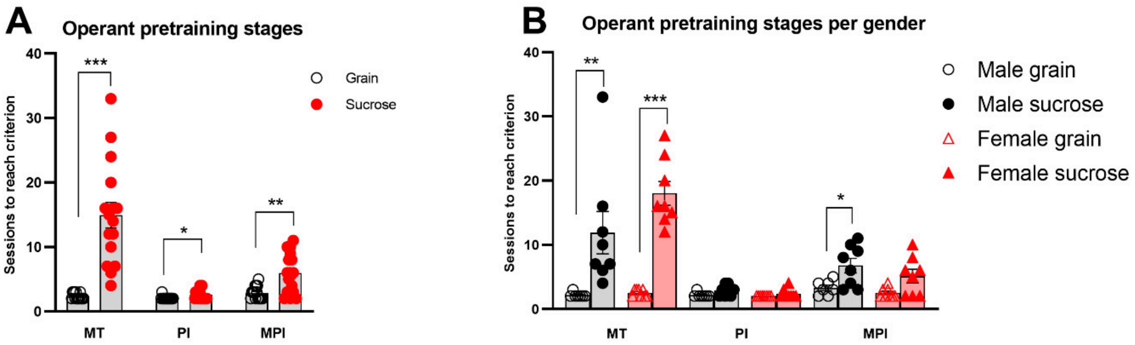

Figure S2. Number of sessions required for male and female LE-sucrose and LE-grain rats to reach criterion during the pretraining stages of the visual discrimination test. (A) Number of sessions required to reach criterion during the pretraining stages for LE rats receiving either grain or sucrose and (B) the same results specified per gender. Values represent the group mean (±SEM). LE-sucrose rats needed more sessions to reach criterion in all training stages: MT ($F_{\text{feeding regime}(1, 27)}$=41.30, p<0.0001), PI ($F_{\text{feeding regime}(1, 27)}$=6.652 p<0.05) and MPI ($F_{\text{feeding regime}(1, 27)}$= 3.26, p<0.01) (B). No significant effects of sex were observed. MT = must touch; PI = punish incorrect; MPI = moving punish incorrect. Asterisks indicate LE grain vs LE sucrose comparisons per feeding regime (for panel A) and per gender of feeding regime (for panel B) ***: p<0.001, **: p<0.01, *:p<0.05.

Figure S3

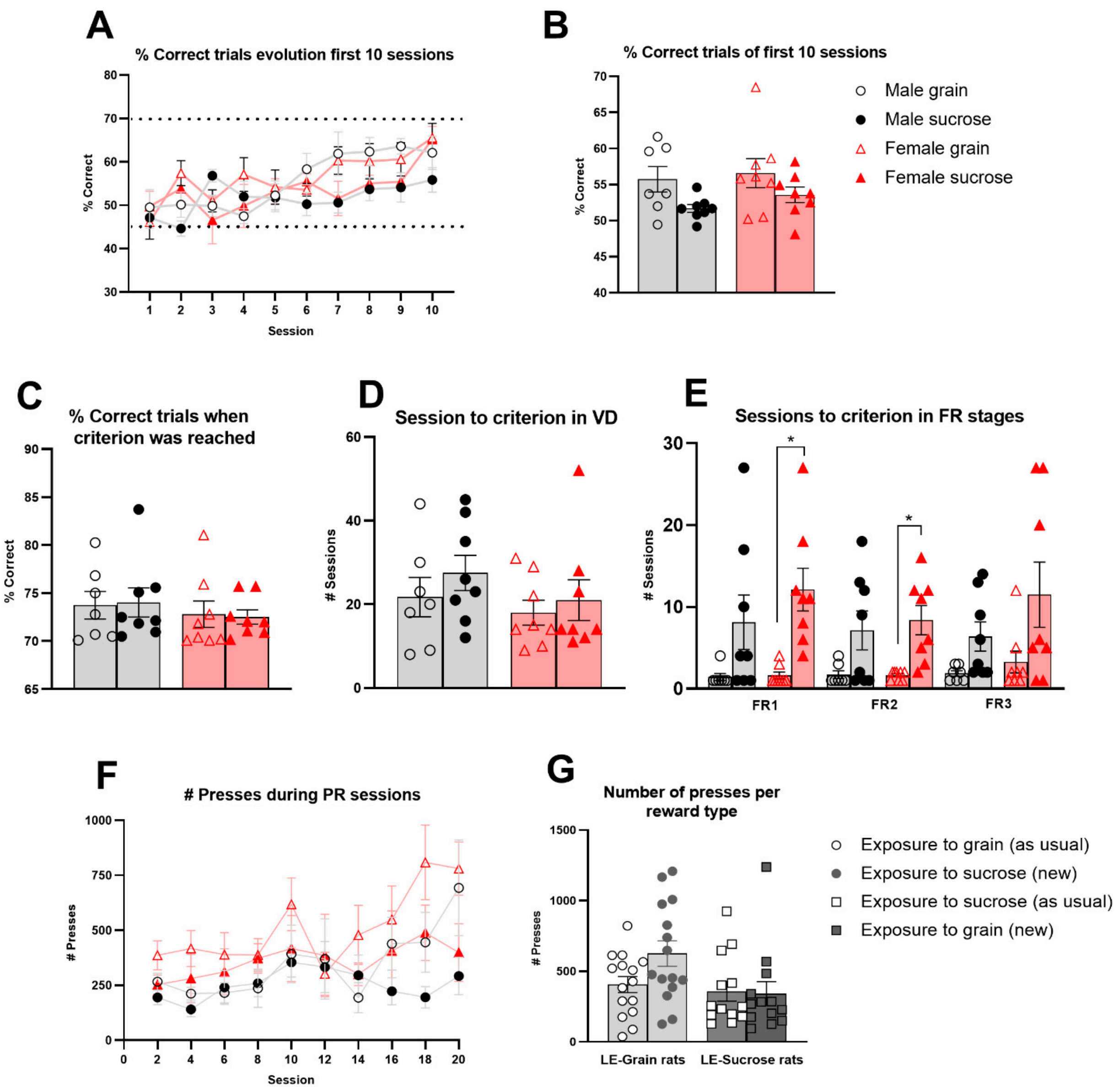

Figure S3. Results of the visual discrimination task, fixed ratio and progressive ratio tasks in male and female LE-sucrose and LE-grain rats. (A) Percentage of correct trials evolution during the 10 first sessions of the visual discrimination (VD) task. (B) Average percentage of correct responding during the first 10 sessions of the VD task. When averaging the correct responses over the first 10 sessions, it was observed that LE grain rats showed a higher percentage of correct responses compared to LE sucrose rats ($F_{feeding\ regime(1,\ 27)}$=5.94, p=0.022), without a significant effect of sex. Additional posthoc analyses indicated a trend towards a higher percentage of correct responses in the male LE grain group compared to the male LE sucrose group (p=0.063). (C) Average percentage of 3 consecutive trials when criterion was reached in which no significant effects were observed between LE grain and LE sucrose rats. (D) Number of sessions required to reach criterion during the VD task was not different between LE grain and LE sucrose rats. (E) Number of sessions required to reach criterion in different Fixed Ratio (FR) stages. The number of sessions required to reach criteria during all FR tasks differed in feeding regime, but not in sex: FR1 ($F_{sex(1,27)}$=0.89, p=n.s.; $F_{feeding\ regime(1,\ 31)}$=15.10, p<0.001); FR2 ($F_{sex(1,27)}$=0.13, p=n.s.; $F_{feeding\ regime(1,\ 27)}$=15.18, p<0.001); FR5 ($F_{sex(1,\ 31)}$=1.89, p=n.s.; $F_{feeding\ regime(1,\ 27)}$=7.26, p<0.05). (F) Average number of correct presses during the 20 progressive ratio (PR) sessions. Only a significant effect of sex was

found in which females performed more correct presses, but no difference in feeding regime was observed ($F_{sex(1, 25)}$=7.03, p<0.05; $F_{feeding\ regime(1, 25)}$=3.29, p=n.s.). (G) Effects of the reward type on the motivation to press for either sucrose or grain pellets. To assess the effect of reward type on motivation, the 3 sessions of the 20 PR sessions (16, 18, 20) with either sucrose or grain as the new reward type, were compared to sessions (15, 17, 19) with the known reward type; the average number of presses for the sucrose reward type during these sessions almost reached significance indicating an overall higher motivation for sucrose rewards ($F_{reward\ type(1, 26)}$=4.201, p=0.051). Values represent the group mean (±SEM). Asterisks indicate LE grain vs LE sucrose comparisons per gender *:p<0.05.

Figure S4

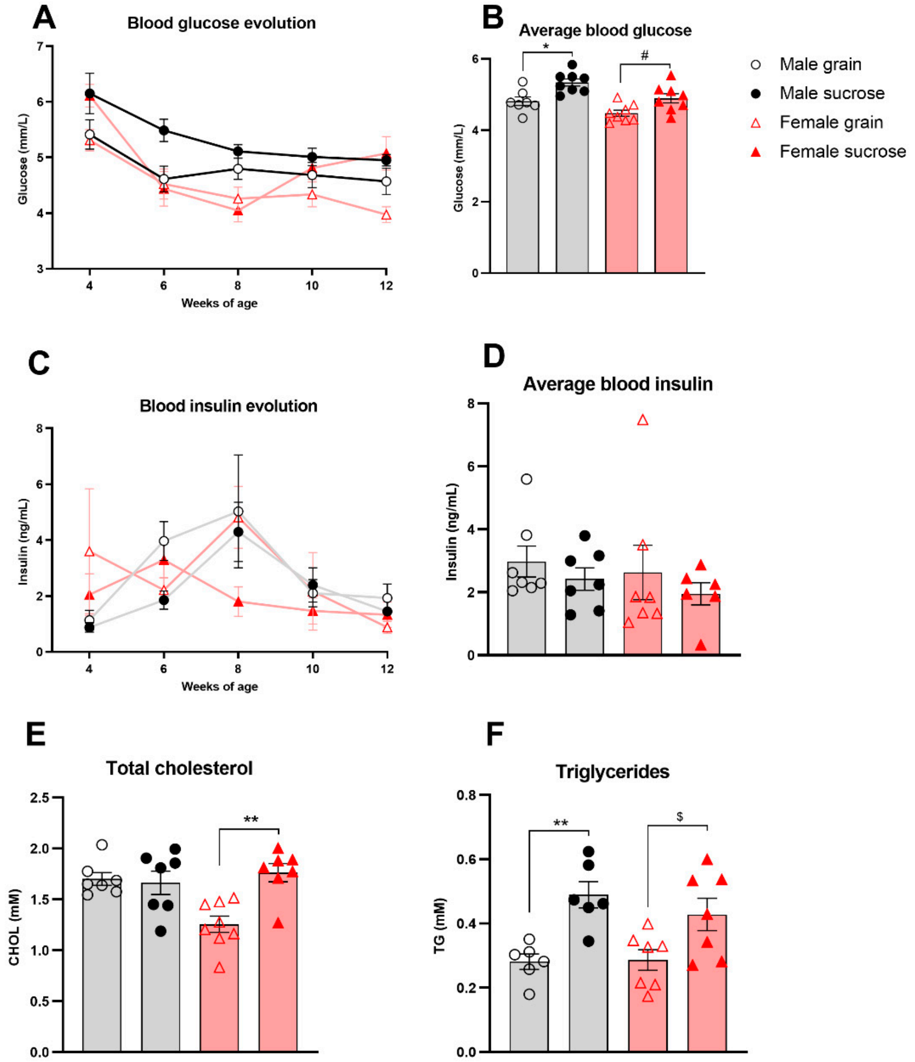

Figure S4. Blood glucose, insulin and lipid levels in male and female LE-sucrose and LE-grain rats. (A, C) Fasted glucose and insulin levels over time at weeks 4, 6, 8, 10 and 12 of age. LE-sucrose rats compared to LE-grain rats presented significantly higher levels of glucose (Glucose: $F_{feeding\ regime(1,\ 27)}$= 17.96, p<0.001; $F_{sex(1,\ 27)}$=12.35, p<0.01;) but not of insulin ($F_{feeding\ regime(1,\ 21)}$=0.06, p=n.s., $F_{sex(1,\ 21)}$=1.47, p=n.s.). (B, D) Average of fasted glucose and insulin levels of weeks 4, 6, 8, 10 and 12 of age. (E) Average total cholesterol levels at week 12 of age. Compared to LE-grain, LE-sucrose rats presented significant higher levels cholesterol ($F_{feeding\ regime\ (1,\ 25)}$=7.03, p<0.05; $F_{sex\ (1,\ 25)}$=3.82, p=n.s.). Furthermore, a significant interaction effect between sex and feeding regime was found for total cholesterol ($F_{(1,\ 25)}$= 9.453, p=0.005). Subsequent multiple comparison analyses indicated that female LE sucrose rats showed higher levels of cholesterol compared to female LE grain rats (p<0.05) while no difference between the male groups was observed. (F) Average total triglyceride levels at week 12 of age. Compared to LE-grain, LE-sucrose rats presented significant higher levels of triglycerides ($F_{feeding\ regime(1,\ 22)}$= 20.06, p<0.001; $F_{sex(1,\ 22)}$= 0.52, p=n.s.) Values represent the mean (±SEM). Asterisks indicate LE grain vs LE sucrose comparisons per gender **: p<0.01, *:p<0.05; #: p=0.055; $:p=0.063.

Figure S5

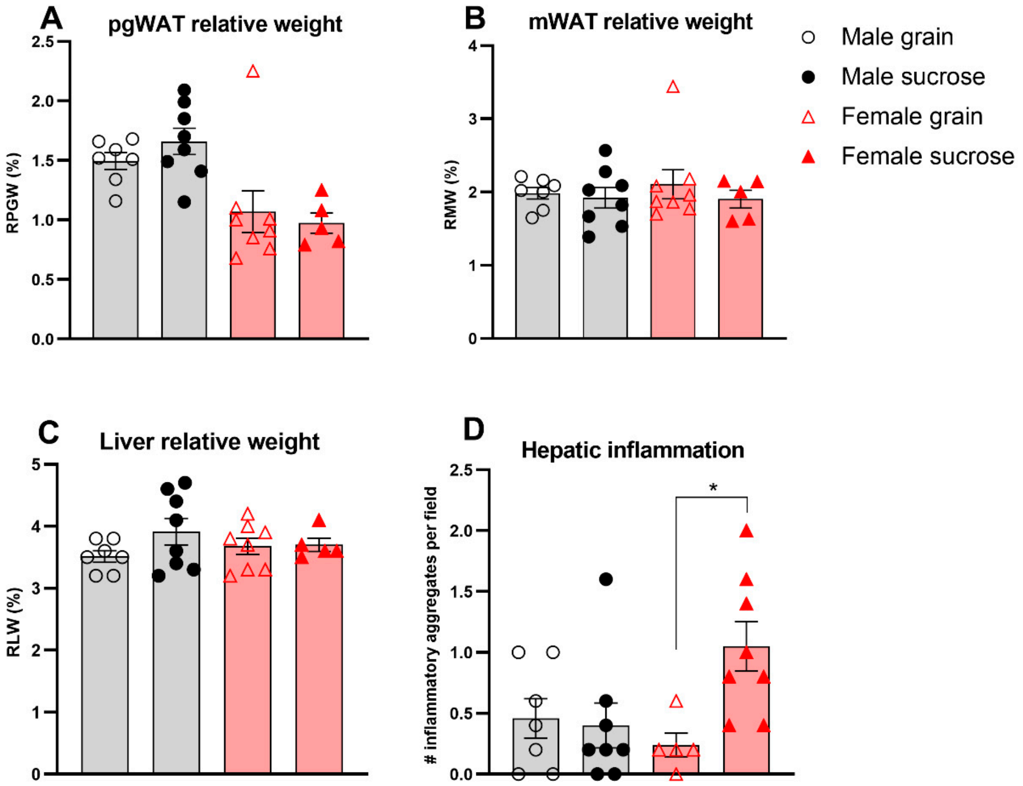

Figure S5. Average relative weights of perigonadal and mesenteric white adipose tissues and liver histopathology in male and female LE-sucrose and LE-grain rats. (A, B) Average relative weights of perigonadal (pgWAT) and mesenteric (mWAT) white adipose tissues. No significant changes were found in relative pgWAT and mWAT in LE-sucrose rats compared LE-grain rats ($F_{\text{feeding regime}(1, 24)}$=0.06, p=n.s.; $F_{\text{feeding regime}(1, 24)}$=0.73, p=n.s.), respectively. (C) Relative liver weights. No difference in relative liver weights was observed ($F_{\text{feeding regime}(1, 24)}$=1.746, p=n.s.). (D) Average of observed inflammatory aggregates within the liver tissue. Interestingly, there was a significantly higher number of immune cell aggregates in livers of female LE-sucrose rats compared to female LE-grain rats ($F_{\text{feeding regime}(1, 24)}$= 5.52, p<0.05). Values represent the group mean (±SEM). Asterisks indicate LE grain vs LE sucrose comparisons per gender *:p<0.05.

Figure S6

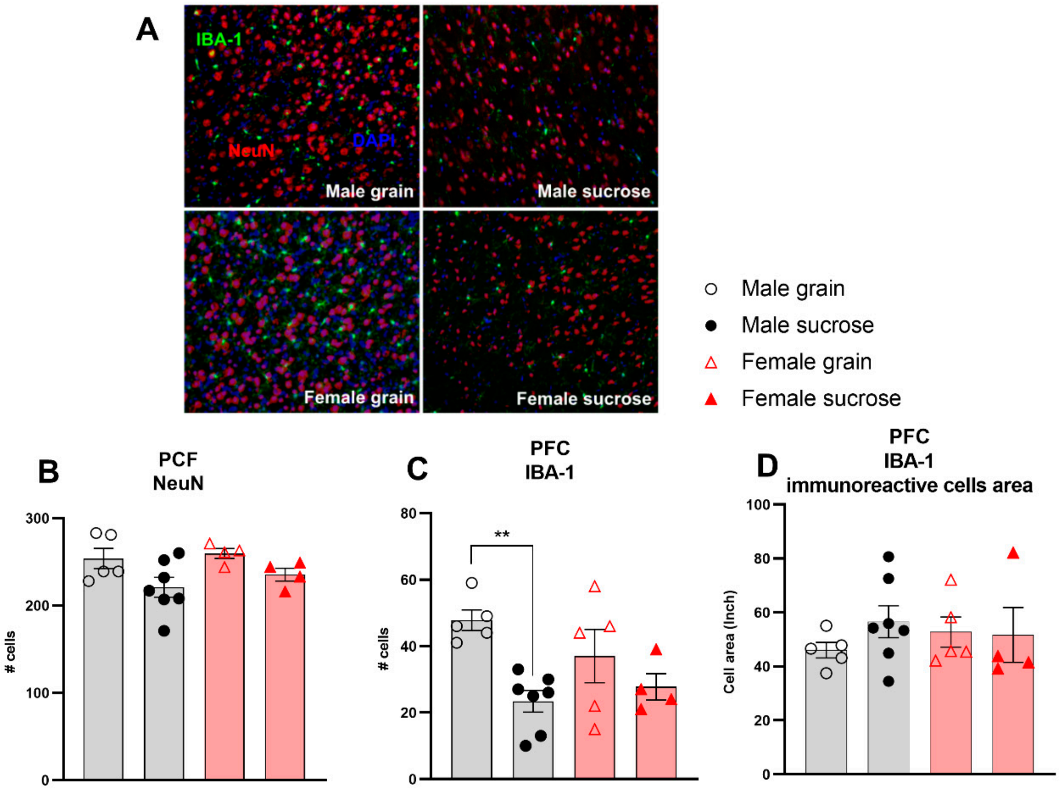

Figure S6. Number of NeuN, IBA-1 positive cells and IBA-1 immunoreactive cells area in the medial prefrontal cortex (PFC) of male and female LE-sucrose and LE-grain rats. (A) Representative images of NeuN and IBA-1 immunoreactive cells. (B) Number of NeuN, (C) Number of IBA-1 cells and (D) immunoreactive cell area of representative IBA-1 cells. Values represent the group mean (±SEM). There was a significant decrease the number of NeuN ($F_{feeding\ regime(1,\ 16)}$= 6.75, p<0.05) and IBA-1 cells ($F_{feeding\ regime(1,\ 17)}$=0.44, p<0.01) in LE-sucrose rats compared to LE-grain rats, but without sex effects. No significant effects of feeding regime and sex was found for the IBA-1 immunoreactive cells area ($F_{genotype(1,\ 17)}$=0.5458, p=n.s.; $F_{sex(1,17)}$=0.01982, p=n.s.). Asterisks indicate LE grain vs LE sucrose comparisons per gender **: p<0.01.

Figure S7

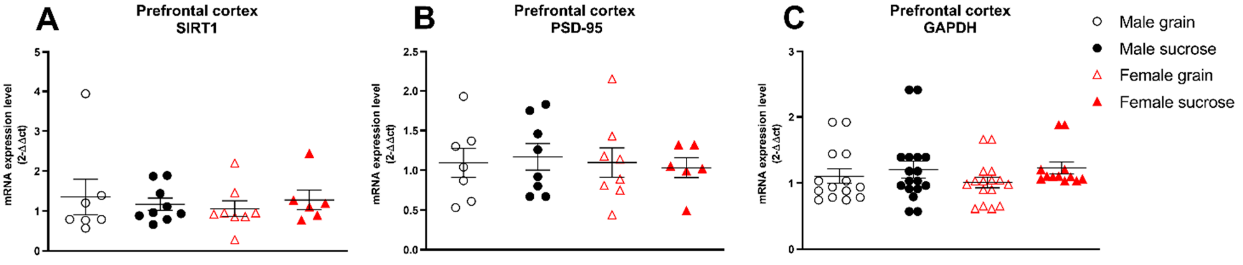

Figure S7. mRNA expression levels of SIRT1 (A), PSD-95 (B) and GADPH (C) in the medial prefrontal cortex of male and female LE-sucrose and LE-grain rats. Values represent the group mean (±SEM). No significant effect of feeding regime was observed (SIRT1: $F_{feeding\ regime(1,\ 26)}$=0.004, p=n.s., $F_{sex(1,30)}$=0.1162, p=n.s.; PSD-95: $F_{feeding\ regime(1,\ 25)=}$0.0008, p=n.s., $F_{sex(1,\ 25)}$=0.1464, p=n.s.; GADPH: $F_{feeding\ regime(1,\ 26)}$=0.9050, p=n.s., $F_{sex(1,\ 26)}$=0.0282, p=n.s.).

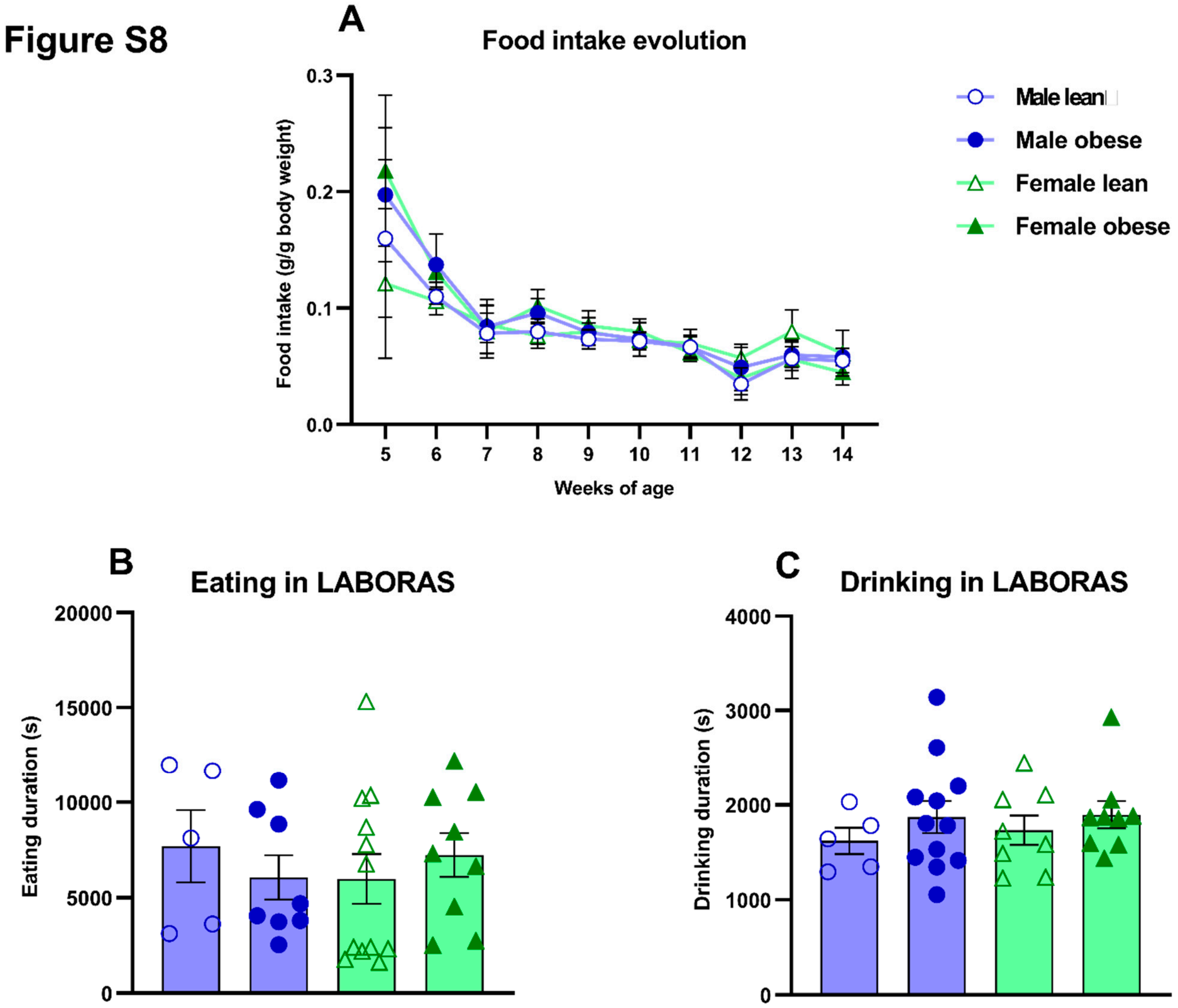

Figure S8. Food intake in male and female ZDF obese and lean rats. (A) Average of weekly food intake *ad libitum*. Values represent the group mean (±SEM). (B, C) Eating and Drinking duration (seconds) in Laboras homecages (Values represent the group mean of 5 sessions of 48 hours each in the Laboras homecage ± SEM).

Figure S9

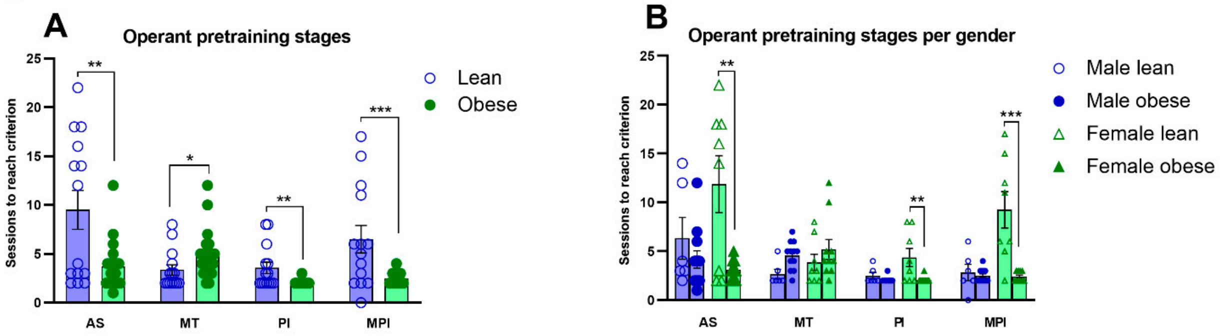

Figure S9. Number of sessions required for male and female obese and lean ZDF rats to reach criterion during the pretraining stages. (A) Number of sessions required to reach criterion during the pretraining stages for lean and obese rats of both sexes together. (B) Number of sessions required to reach criterion during the pretraining stages, specified per gender and genotype. Values represent the group mean (±SEM). AS = autoshaping; MT = must touch; PI = punish incorrect; MPI = moving punish incorrect. Asterisks indicate lean vs obese group comparisons (for panel A) and per gender (for panel B) ***: $p<0.001$, **: $p<0.01$, *:$p<0.05$.

Figure S10

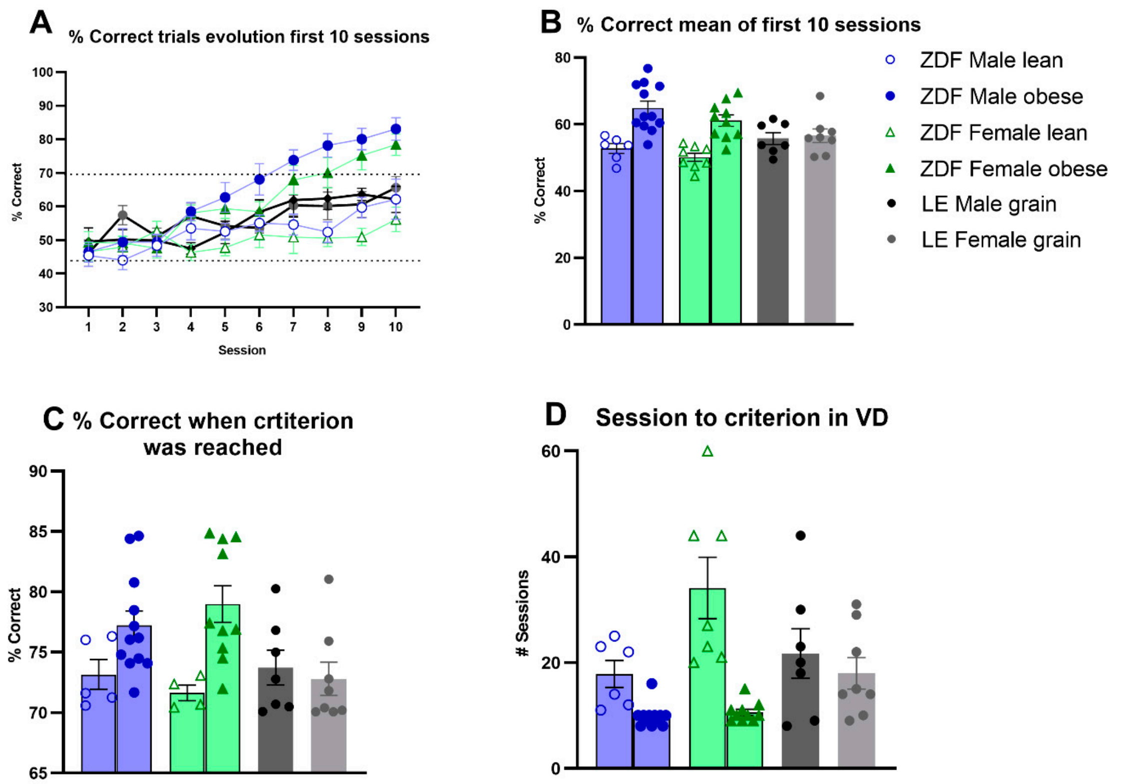

Figure S10. Cognitive performance of male and female obese and lean ZDF rats and healthy LE rats under the same feeding regime. (A) The evolution of percentage of correct trials during the first 10 sessions of the visual discrimination (VD) task. A significant interaction was observed between group x session ($F_{group\ x\ session(18,\ 378)}$=4.85, p<0.001) and a main effect of group ($F_{group(2,\ 42)}$=16.47, p<0.001). Subsequent analyses per group indicated that the ZDF obese group showed a significantly higher percentage of correct responding compared to both the ZDF lean group and the LE grain group. (B) Average values of the percentages of the correct trials during the first 10 VD sessions. We observed a main effect of group only ($F_{group(2,\ 45)}$=20.00, p<0.001). Post-hoc analyses indicated that all three groups differed from each other, p<0.05 (when not taking gender into account). Specified per gender, showed that male LE grain rats did not differ from the ZDF lean rats, but that ZDF obese rats performed better compared to the LE grain males. For females, LE grain rats did not differ from the ZDF obese group (p=0.089), but performed significantly better compared to the ZDF lean females. (C) Average percentage of correct trials of the 3 consecutive trials when task criterion was reached. A main effect of group was observed ($F_{group(2,\ 40)}$=9.92, p<0.001). Post-hoc analyses indicated that LE grain rats did not differ from lean ZDF rats, but had a significantly lower level of correct percentage compared to ZDF obese rats (when not taking gender into account). Specified per gender, showed that male groups did not differ among each other, and for females, LE grain rats performed significantly worse compared to ZDF obese rats. (D) Number of sessions required to reach criterion during the VD task. A main effect of group ($F_{group(2,\ 44)}$=12.72, p<0.001) and a main interaction effect between group and sex was observed ($F_{group\ x\ sex(2,\ 44)}$=3.97, p=0.026). Post-hoc analyses indicated that LE grain rats did not differ from lean ZDF rats, but needed significantly more sessions to reach criterion compared to ZDF obese rats (when not taking gender into account). Specified per gender, showed that male LE grain rats needed more sessions compared to ZDF obese males. For females, the LE grain rats, needed significantly less sessions compared to ZDF lean rats, but did not differ from ZDF obese rats. Values during the VD task represent the group mean (±SEM).
